# Precipitous intussusception with anal protrusion and complete overt rectal prolapse presenting with intestinal obstruction and an associated rectal adenoma in a young man: a case report

**DOI:** 10.1186/1756-0500-6-401

**Published:** 2013-10-05

**Authors:** Peter A Ongom, Robert L Lukande

**Affiliations:** 1 Colorectal Surgery Unit, Department of Surgery, School of Medicine, Makerere College of Health Sciences, Makerere University, P.O. Box 7072, Kampala, Uganda; 2 Histopathology Unit, Department of Pathology, School of Biomedical Sciences, Makerere College of Health Sciences, Makerere University, P.O. Box 7072, Kampala, Uganda

**Keywords:** Intussusception, Complete overt rectal prolapse, Adenoma, Anal protrusion, Intestinal obstruction

## Abstract

**Background:**

Intestinal obstruction secondary to intussusception, occurring simultaneously with complete rectal prolapse, is an unusual entity among young adults. When it occurs the intussusceptum may protrude per anus. Few cases are cited in literature; each with a unique clinical presentation. There is apparently no uniform trend in its clinical and pathological picture.

**Case presentation:**

A 38-year-old, African-Ugandan man presented with sudden occurrence of rectal prolapse for one day. He had otherwise been in good health. Symptoms were precipitous. A clinical diagnosis of intussusception of the lower gut with rectal prolapse, and intestinal obstruction, was made. The intussusception was found to have a polyp as the ‘lead point’. He was treated by manual reduction of the intussusception and the prolapse under general anesthesia. Histopathologic examination of the polyp showed it to be an adenoma. Definitive surgical treatment of the patient was not completed due to socioeconomic challenges.

**Conclusions:**

Rectal prolapse and intussusception are commonly childhood conditions. Rectal prolapse alone is commoner in the middle-aged and elderly; females in particular. The finding of this combined clinical entity in a young, adult male is therefore a unique condition with an unusual presentation. It is the first case of its kind reported in East Africa. It is also an example of an adenoma constituting a ‘lead point’ for an intussusception at the gastrointestinal tract’s terminus. Even in the presence of a pre-existing adenoma, a relatively common lesion, other differential diagnoses acting as ‘lead points’ ought to be considered in perspective. This characteristic, along with other features described in this case, is useful knowledge for colorectal surgeons, general surgeons, gastrointestinal pathologists, and gastroenterologists given their involvement in the diagnosis and management of anorectal disease of peculiar presentation.

## Background

Intussusception is the ‘telescoping’ of a segment of the intestine into the lumen of another immediately adjacent segment. Among adults it is relatively rare, constituting less than 5 percent of intussusception cases, with a male to female ratio of 1.8 to 1 [1]. In resource-rich countries, the incidence is 2 to 3 per 1,000,000 per year, representing 1 to 3 percent of all cases of intestinal obstruction [2] and constituting less than 1 percent of hospital admissions [3]. The entity of anal protrusion of an intussusception is well documented among infants with a prevalence of 29 percent of all cases of intussusception in one series report [4].

Rectal prolapse is the ‘slipping’ or ‘falling out-of-place’ of the rectum. This may involve its mucosa (partial prolapse) or the full thickness of all its layers (complete prolapse). It is also described as ‘revealed’ (overt) or ‘hidden’ (covert). The terminal 12 to 15 cm of gut constitutes the affected area. Typically the condition is commoner in females (80 to 90 percent) compared to males [5], and among the elderly compared to younger age groups. Intussusception with concurrent rectal prolapse is rare.

Generally, intussusception is not usually associated with a demonstrable cause (idiopathic). This particularly is characteristic of childhood intussusception; the commoner type. In adults, there is a demonstrable cause (with a ‘lead point’) in the majority of cases, usually an intra-luminal neoplasm. A number of studies point to a 70 to 90 percent existence of an underlying gut pathological cause [2]. These are mainly polyps and colonic malignancies.

Our case features an intussusception with a rectal adenoma and a concurrent complete rectal prolapse protruding per anus. It can also be described as an anal protrusion of an intussusception, alongside a rectal prolapse. This simultaneous occurrence culminated in acute intestinal obstruction. The entire presentation is rare and unique. The commonest lesions constituting a ‘lead point’ are colonic malignant tumors which occur in up to 60 percent of cases [2]. Benign tumors constitute the majority of the rest of the causes. Of these, adenomas are the commonest [3,6].

## Case presentation

Our patient is a 38-year-old African-Ugandan man, of Bantu ethnicity. He presented to the Emergency Department of Mulago National Referral and Teaching Hospital with complaints of protrusion of a mass per anus, abdominal pain, low-back pain and vomiting for one day. These were associated with passage of loose, bloody and mucoid stool. Moments prior to the onset of these symptoms, he felt the ‘normal’ urge to empty his bowels. Upon bearing down he was unable to empty his bowels normally and found that he had to exert extra effort. He reported a sudden protrusion of a mass consisting of his ‘gut’ through his anus, accompanied by excruciating anal pain. Moments later the protruding mass began exuding a bloody and mucoid discharge. This was followed by severe low-back pain, generalised abdominal pain, vomiting and constipation. Because of these symptoms, he was first taken to the local district hospital from where he was referred to the National Referral Hospital without any intervention. The rest of his medical history was unremarkable.

Prior to all these events he was reportedly in good health. He first had overt haematochezia three years ago, and had had three episodes over this period. On each of these occasions there was an accompanying loose, mucoid stool motion. There was also an unusually strong effort exerted to effect voiding of stool. During those periods he also had on and off low back pain, which increased in intensity with each subsequent episode. The pain was relieved with analgesics and bed rest. He partly attributed this pain to his occupation of subsistence fishing; a laborious undertaking. He had no history of protrusion of a mass per anus previously, even on heavy exertion. He has had tenesmus and a feeling of incomplete bowel emptying on a few occasions. However, he had neither a history of abdominal swelling nor one of significant weight loss.

On physical examination, he was in pain, alert and maintained a knee-elbow posture. He was afebrile (temperature, 37°C), moderately dehydrated, but was not pale (hemoglobin level was 12.7 gm/dL), and had no significant peripheral lymphadenopathy. His radial pulse was 98 beats/minute, regular and synchronous with other pulses, and his blood pressure was 110/70 mmHg. He had a moderately distended abdomen that moved with respiration. Further examination was done in the operating theatre with the patient under sedation. Blood grouping was also done. An intravenous line was instituted for fluid therapy and antibiotics were administered (1 gm, ceftriaxone and 500 mg, metronidazole).

**Figure 1:**
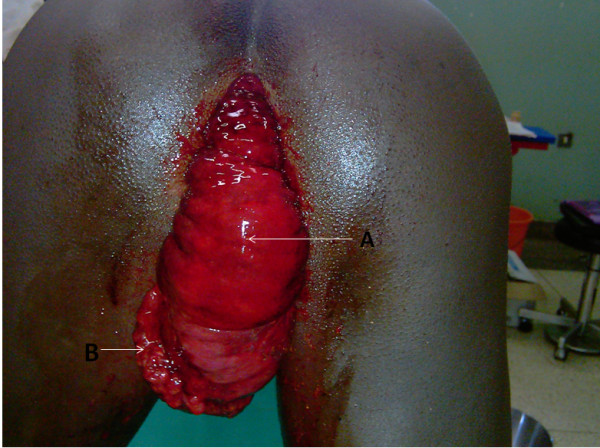
**Photograph of mass protruding per anus.** The photograph shows anal protrusion of a mass per anus. It consists of a prolapsed intussusception of the terminal lower gut; anal canal, rectum and terminal sigmoid colon. The patient is in a knee-elbow position. Labeled areas are: **A** – Oedeamatous rectal mucosa (intussusceptum); **B** – Sessile polyp (adenoma) on the anterior aspect of the lower 1/3 of the rectum.

Continued physical examination under sedation showed no visible peristalsis and no obvious masses palpable per abdomen. His liver, spleen, and kidneys were not palpable, and a tympanic percussion note was heard throughout the abdomen. On auscultation, his bowel sounds were increased in frequency and pitch. The rectal examination revealed a large, sausage-shaped mass protruding from the anal verge consisting of the anal canal, rectum and apparently, parts of the terminal sigmoid colon. It was 15–20 cm in length, with an anterior convexity. The lower antero-inferior aspect had a sessile, polypoid growth from the mucosa (Figure 1). It was covered with a bloody, mucoid exudate (Figure 1). There was also extensive oedema and areas of haemorrhagic foci, but no formed stool. The gut was viable with no signs of gangrene. The rest of his systemic examination was unremarkable.

A diagnosis of precipitous intussusception with rectal prolapse, causing intestinal obstruction, was made. General anesthesia was induced with the patient placed in the lithotomy position. The protruding mass was gently raised and a digital examination of the anal verge and canal attempted. The anal canal admitted only a finger tip anteriorly, and approximately 2 cm of the examining digit posteriorly. An incisional biopsy of the anterior polypoid growth (‘lead point’) was taken. Gentle but steady pressure was applied to the mass in a distal-to-proximal direction. Gradually, the mass was reduced completely over a period of about 5 minutes. At the end of the manoeuvre there was passage of flatus per anus and a reduction in abdominal distension. A digital rectal exam confirmed complete reduction of the prolapse and the intussusception, with the polyp palpable in the anterior aspect of the lower third of the rectum (5 cm from the anal verge). Histopathologic examination of the biopsy revealed an adenoma.

## Discussion

We have highlighted that adult intussusception causing intestinal obstruction and occurring simultaneously with complete rectal prolapse is uncommon. But that our patient presented with this combination of disease entities. For the intussusception component, his age of 38 years is particularly uncharacteristic, while his gender is less atypical [1,4]. Intussusception in adults may present with acute, sub-acute, or chronic non-specific features, making its diagnosis occasionally difficult [7]. This patient’s intussusception had a precipitous (‘very’ acute) onset and was overt, with protrusion through the anal verge; the reason for his admission. The complete rectal prolapse component presents with a chronic clinical course – complaints of low backache following strenuous activity over a period of three years.

Typical intussusception, as seen in childhood, usually presents with a classic triad of symptoms and signs: cramp-like abdominal pain, a palpable sausage-shaped mass (mainly in the right upper quadrant) and currant jelly stools [8]. Adults do not commonly have all these features. Complete rectal prolapse usually presents with a protruding anal mass (overt) that may be reducible spontaneously or manually, and is largely painless. Commonly, there is a history of chronic constipation and a progressive increase in the size of the prolapse with time [9]. A practical clinical challenge is the distinction between complete (full-thickness) rectal prolapse and partial (mucosal) rectal prolapse [10]. This is not straight forward in many cases.

An explanation of his symptoms is derived from the anatomical abnormalities related to rectal prolapse, and the predisposing pathology associated with intussusception. Anatomical features associated with rectal prolapse include: an ‘intussusception’ per se; a deep rectovesical pouch; absent fixation of the rectum to the sacrum; a redundant rectum and sigmoid colon; weakness of the pelvic floor and/or anal sphincter muscles; and possibly, the presence of a rectocele [11]. One or more of these is present in the event of a prolapse. Chronic low back pain with previous episodes of straining on defaecation is a feature in any of these conditions. A rectal exam of the anal protruding mass, with very limited or no admittance of the examining digit, classically defines the rectal prolapse entity [12]. However, one can argue that even the limited admittance of an examining digit qualifies it as an intussusception, thus the description of a combined clinical condition.

A history of haematochezia, tenesmus and a feeling of incomplete voiding explains the pathophysiology of an intussusception secondary to an underlying lesion (‘lead point’); in this case, an adenomatous polyp. This polyp caused on-and-off bleeding and a mass effect, presenting as tenesmus and a real or apparent feeling of incomplete rectal emptying upon voiding. A chronic clinical course ensued. The final protrusion of the polyp with the intussusceptum and rectal prolapse upon strongly bearing down happened to all occur in a precipitous fashion causing severe abdominal and low back pain, abdominal distension and complete constipation [7]. This constituted an acute intestinal obstruction. The antecedent oedema and bloody, mucoid exudate are due to venous obstruction, inflammation and mucosal haemorrhage [13]. These features compare well with intussusception in a vulnerable age group (childhood); up to 8 percent have a prolapsing intussusception [14]. The most common presenting features remain rectal bleeding, an abdominal mass and abdominal pain.

**Figure 2:**
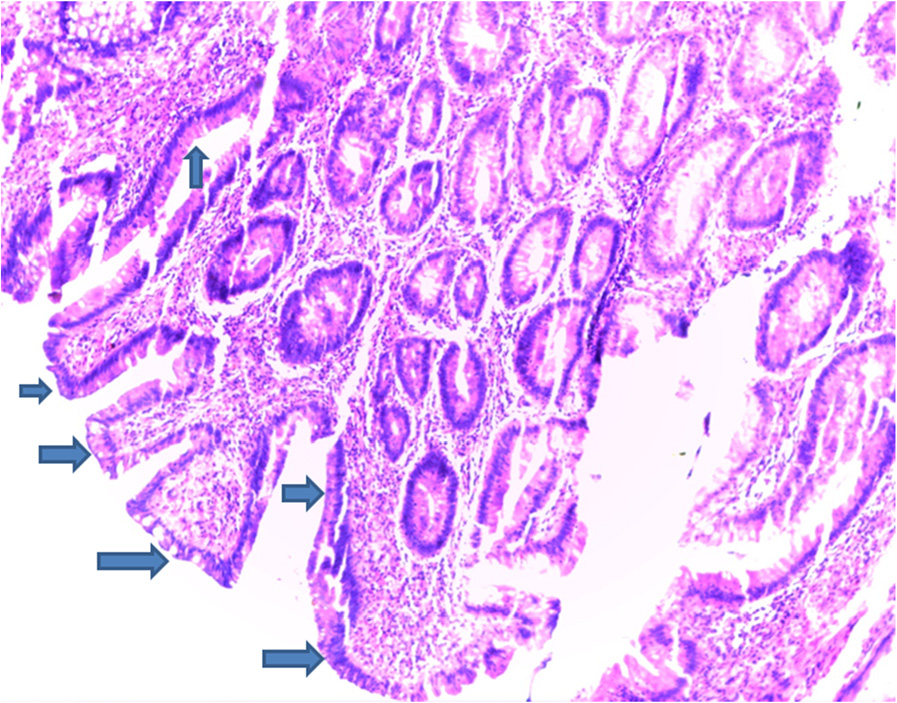
**Photomicrograph of the rectal adenoma.** This shows proliferating, crowded, hypercellular colonic tubules (arrows). It constitutes a tubular adenoma (magnification x 10, objective lens).

**Figure 3:**
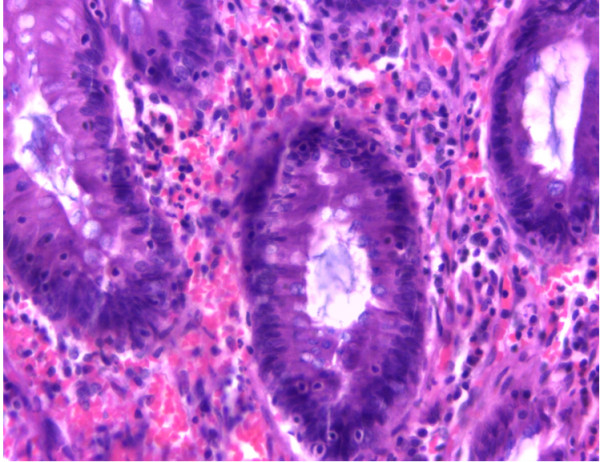
**Photomicrograph of the rectal adenoma at higher magnification.** Hematoxylin and eosin stained section of gut showing low-grade dysplasia; no architectural complexity (magnification × 20, objective lens).

A biopsy of the polyp was essential to determine its histopathologic characteristics. In this case it was a tubular adenoma (Figure 2), contrary to the majority of ‘lead points’ for intussusception (most are adenocarcinomas) [8]. Some other pathological lesions give a similar spectrum of clinical presentation that ought to be ruled out. Significant among these is the rectal prolapse syndrome [15]. This refers to a number of anorectal conditions that include: cap polyposis, hamartomatous inverted polyps, inflammatory cloacogenic polyps, localised colitis cystica profunda and solitary rectal ulcer syndrome. These provide us with the differential diagnosis of rectal polyps. They have typical anatomical locations (often around the same site as in our patient) and share a histomorphological overlap in clinical presentation. Each of these conditions may present with features of a rectal prolapse, with or without an intussusception. Nevertheless, most definitive diagnoses of specific lesions are made through routine histopathological examination. All these differential diagnoses will exhibit features different from the characteristic adenoma picture portrayed (Figures 2, 3).

The diagnosis in our case was largely clinical, and the ‘lead point’ was defined histopathologically. As the patient presented with an acute intestinal obstruction, no other special investigations like plain radiographs, ultrasound scans (USS) or computerized tomography (CT) were done as the nature of his symptoms and signs meant that he could only maintain a knee-elbow position and the immediate priority then was relief of the obstruction by reduction of the intussusception and prolapse. In cases of uncertainty, CT scans are the most efficient diagnostic investigation with an accuracy of 58 to 100 per cent, followed by USS [1,2].

Management of this patient was aimed at relieving the intestinal obstruction, followed later by definitive treatment of the cause. To relieve the obstruction we first adequately resuscitated him, after which thorough examination and reduction of the mass was done under general anaesthesia [7]. All gut was found viable and successfully reduced. Unfortunately, definitive surgery for the adenoma and rectal prolapse was not done due to our patient’s socioeconomic challenges. We had a plan of management that included: i) conducting barium enema studies – to assess the sigmoid colon for possible ‘redundancy’ (a common predisposing factor for prolapse), and for other filling defects (possibly additional polyps); ii) colonoscopy – to check for the presence of synchronous tumours, and if found biopsies taken; and iii) performing definitive surgery to treat the prolapse and the adenoma. The planned surgical procedure was slated to be sigmoid colectomy, if the sigmoid colon was found to be abnormal, along with a trans-abdominal rectopexy and excision of the adenoma. However, following the great relief of symptoms after the emergency measures taken, involving reduction of the prolapse and overcoming the intestinal obstruction, coupled with him being the ‘bread winner’ at home, he insisted on leaving hospital with a commitment to return for the planned course of management. He did not return.

## Conclusion

Intussusception and complete rectal prolapse are well described and documented disease entities. While complete rectal prolapse is common among children and older adults, particularly females, it is uncommon among young, adult males. The dividing line between these two entities is clearly defined clinically, but may have simultaneous and overlapping presentation.

Complete rectal prolapse is a top differential diagnosis of intussusception with prolapse per anus. It is of paramount importance for one to always assume the presence of an intussusception, even when it may mimic complete rectal prolapse alone, especially with a precipitous presentation as is evidenced in our patient. This is mainly because intussusception causes acute intestinal obstruction whose sequelae may be life threatening, especially gut strangulation with gangrene. This makes it a surgical emergency. On top of this, intussusception in adults is commonly related to other underlying pathology. There is usually a ‘lead point’; an adenoma as in our case. More commonly however the ‘lead point’ is an adenocarcinoma, whose presence paints an even grimmer picture. In contrast, complete rectal prolapse alone tends to have a gradual course of progression. It is largely benign and not associated with a bad outcome in the short run.

## Consent

Written, informed consent was obtained from the patient for publication of this case report and any accompanying images. A copy of the written consent is available for review by the Editor-in-Chief of this journal.

## Competing interests

The authors declare that they have no competing interests.

## Authors’ contributions

PAO managed our patient peri-operatively and wrote the manuscript. RLL co-wrote the manuscript, and examined, analysed and described the histopathological slides. Both authors read and approved the final manuscript.
